# The double burden: type 1 diabetes and heart failure—a comprehensive review

**DOI:** 10.1186/s12933-024-02136-y

**Published:** 2024-02-12

**Authors:** María Teresa Julián, Alejandra Pérez-Montes de Oca, Josep Julve, Nuria Alonso

**Affiliations:** 1 Department of Endocrinology and Nutrition, Hospital Germans Trias i Pujol, Badalona, Spain; 2 Department of Medicine, Universitat Autònoma de Barcelona, Barcelona, Spain; 3 Institut d’Investigació Biomèdica Sant Pau (IIB Sant Pau), Barcelona, Spain; 4 Center for Biomedical Research on Diabetes and Associated Metabolic Diseases (CIBERDEM), Instituto de Salud Carlos III, Madrid, Spain

**Keywords:** Heart failure, Diabetes mellitus, Type 1 diabetes, Diabetic cardiomyopathy

## Abstract

Heart failure (HF) is increasing at an alarming rate, primary due to the rising in aging, obesity and diabetes. Notably, individuals with type 1 diabetes (T1D) face a significantly elevated risk of HF, leading to more hospitalizations and increased case fatality rates. Several risk factors contribute to HF in T1D, including poor glycemic control, female gender, smoking, hypertension, elevated BMI, and albuminuria. However, early and intensive glycemic control can mitigate the long-term risk of HF in individuals with T1D. The pathophysiology of diabetes-associated HF is complex and multifactorial, and the underlying mechanisms in T1D remain incompletely elucidated. In terms of treatment, much of the evidence comes from type 2 diabetes (T2D) populations, so applying it to T1D requires caution. Sodium-glucose cotransporter 2 inhibitors have shown benefits in HF outcomes, even in non-diabetic populations. However, most of the information about HF and the evidence from cardiovascular safety trials related to glucose lowering medications refer to T2D. Glycemic control is key, but the link between hypoglycemia and HF hospitalization risk requires further study. Glycemic variability, common in T1D, is an independent HF risk factor. Technological advances offer the potential to improve glycemic control, including glycemic variability, and may play a role in preventing HF. In summary, HF in T1D is a complex challenge with unique dimensions. This review focuses on HF in individuals with T1D, exploring its epidemiology, risk factors, pathophysiology, diagnosis and treatment, which is crucial for developing tailored prevention and management strategies for this population.

## Introduction

Heart failure (HF) currently represents a global health problem due to the significant levels of morbidity and mortality associated with it [1, 2]. Although the treatment of HF has improved in recent years, its prevalence and incidence have increased, leading to a substantial number of hospital admissions, progressive deterioration in the quality of life, and increased mortality. It is well established that diabetes mellitus (DM) is a significant risk factor for the development of heart disease, including HF [3]. Numerous epidemiological studies have established that diabetes is independently associated with the risk of developing HF [3–5]. Importantly, recent findings have revealed that among individuals with DM, especially those with type 2 diabetes (T2D), HF is increasingly becoming the primary manifestation of cardiovascular conditions, overtaking atherosclerotic diseases in this regard [6]. Indeed, the rising prevalence of DM worldwide and the aging of the world’s population have led to the emergence of a significant problem associated with diabetes-related HF [7, 8]. The relationship between DM and HF is complex and multifactorial, and several mechanisms have been implicated. Diabetes increases the risk of HF regardless of classical cardiovascular risk factors such as hypertension or coronary heart disease. While the existence of distinct diabetic cardiomyopathy (DCM) remains a subject of debate, numerous experimental and preclinical studies have shown that hyperglycemia results in structural, functional, metabolic, and hemodynamic alterations in the myocardium [9, 10].

In recent years, there has been growing interest in HF due to the development of new therapies, including glucose-lowering medications, such as sodium-glucose cotransporter 2 inhibitors (SGLT2), which have demonstrated significant cardioprotective effects, leading to notable improvements in HF symptoms, reduced hospitalizations rates, and decreased mortality [11]. Nonetheless, most of the knowledge concerning HF and the evidence from cardiovascular safety trials involving antidiabetic drugs refer to T2D.

Type 1 diabetes (T1D) is a chronic autoimmune disorder characterized by the destruction of insulin-producing beta cells in the pancreas. Similar to what occurs in T2D, cardiovascular disease, which includes HF, emerges as a long-term complication in T1D [12]. Recent epidemiological findings have shown an increasing prevalence of HF in individuals with T1D, potentially linked to a growing population of older individuals with long-standing T1D. However, HF in patients with T1D has not been studied as comprehensively as in patients with T2D. Understanding the complex mechanisms that link T1D and HF is crucial for the development of effective strategies for prevention and management. In this review, our primary focus will be on examining the evidence regarding heart failure in individuals with T1D, with particular attention paid to aspects such as epidemiology, risk factors, pathophysiology, and treatment options.

## Methodology

We conducted a systematic search on the electronic database PubMed to look for relevant articles based on the research question. Papers were selected for inclusion in the present review according to their relevance, as judged by the authors. As a literature review, no ethics committee approval was needed.

## Epidemiology, risk factors and prognosis

In developed countries, HF affects approximately 1–2% of the adult population [6, 13], and in elderly individuals, the prevalence can rise to more than 10% [14]. Data from observational [15] and systematic studies [16] suggest a significant increase in the incidence rate of HF in subjects with T1D (Table 1) and a high risk of hospitalization due to HF among individuals with T1D, a risk that may quadruple that of the general population [17, 18]. A 10-year retrospective study by McAllister et al. [17] found 1313 occurrences of HF among patients with T1D of more than 3.25 million adults without DM, T2D, and T1D. The crude incidence rate of HF hospitalization in the T1D group was 5.6 per 1000 person-years, compared with 2.4 cases in individuals without DM and 12.4 cases in those with T2D. Patients with T1D had a higher case fatality rate than people without DM and the difference was bigger in men (OR, 1.91; 95% CI, 1.68–2.18) than in women (OR, 1.31; 95% CI, 1.05–1.65) [17].

**Table 1:** Studies examining the association between type 1 diabetes mellitus and heart failure

Study/Author	Design	Follow-up (years)	Subjects with T1D*	Results
Haji et al. 2023 [17]	Meta-analysis	From 1–12	61,885	RR 3.4 (95% CI, 2.71–4.26)
Giménez‐Pérez et al. 2023 [179]	Retrospective cohort	6	8412	The occurrence of HF was 14.4%. In women > 65y HF was the most frequent event (40.5%)
Chadalavada et al. 2021 [25]	Prospective cohort	8	2626	RR 2.92 (95% CI, 2.57–3.32)
Avogaro et al. 2020 [16]	Meta-analysis	11 ± 3	160,096	IRR 2.9 (95% CI, 2.11–3.99)
Cai et al. 2020 [20]	Meta-analysis	From 4.5–24	166,027	RR 4.3 (95% CI, 3.54–5.19)
Larsson et al. 2018 [180]	Prospective cohort	17	247	RR 2.7 (95% CI, 1.76–4.09)
McAllister et al. 2018 [18]	Retrospective cohort	10	25,610	IRR 2.32 (95% CI, 2.20–2.45)
Rawshani et al. 2018 [26]	Retrospective cohort	10	27,195	IRR 5.39 (95% CI, 0.46–62.80)
Rosengren et al. 2015 [19]	Prospective cohort	7.9	33,402	IRR 4.12 (95% CI, 3.80–4.47)
Lind et al. 2011 [23]	Prospective cohort	9	20,985	IRR 3.48 (95% CI, 3.17–3.83)

*T1D* type 1 diabetes, *CI* confidence interval, *RR* relative risk, *IRR* incidence rate ratio, *HF* heart failure

^*^patient-years

Furthermore, a recent meta-analysis investigated the risk of HF in individuals with T1D compared to those without DM. They reviewed four studies, with follow-up periods ranging from 1 to 12 years, and found that there had been a total of 1378 HF events among individuals with T1D, 3993 among those with T2D, and 18,945 among the controls. The incidence rate of HF per 1000 person-years was 5.8 for T1D, 10.0 for T2D, and 2.3 for controls. T1D patients had a three-fold higher risk of HF compared to controls (RR 3.4) and this risk was approximately five times higher in women with T1D (RR 4.9) compared to men (RR 3.0) [16]. Moreover, a separate systematic review that analyzed six observational studies, found that the HF incidence rate in T1D patients was also three times higher than in healthy controls (*p* < 0.001). The analysis indicated a correlation between HF risk and the age of T1D patients, suggesting that careful monitoring of HF risk factors is crucial, mainly since early diabetes onset may be a significant factor in reducing HF risk in this population. For every 10 years of disease duration, there was a slight increase of 0.003 in the Incidence Rate (IR) of HF, although this trend did not reach statistical significance (*p* value = 0.07) [15]. Additionally, this elevated risk of developing HF for T1D individuals was found to be even higher, approximately four times so, in a meta-analysis aimed at investigating the association between T1D and cardiovascular disease (CVD) (Table 1). The study also noted an elevated risk of HF among females with T1D [19].

Regarding the different phenotypes based on left ventricular ejection fraction (LVEF) (heart failure with reduced ejection fraction [HFrEF], heart failure with mid-range ejection fraction [HFmrEF], heart failure with preserved ejection fraction [HFpEF], the available data is very limited. In a 7-year prospective study involving individuals with long-standing T1D, the overall prevalence of HF at the end of the follow-up period was 3.7%. Among the patients with HF, 85% exhibited HFpEF (defined by LVEF ≥ 50%), while the remaining 15% had HFrEF (defined by LVEF < 50%) [20]. Similar to other conditions, there is a lack of data regarding the prevalence of HFmrEF (defined as LVEF 40–49%) because most epidemiological studies, including the aforementioned one, have categorized HF patients into two groups using an LVEF cutoff value of 50%. Moreover, a recent study included 154 patients with T1D and myocardial dysfunction from the Thousand & 1 study as a comparison subgroup. Although this study assessed LVEF in individuals with T1D, it primarily focused on subjects without known heart disease. Notably, the study only reported the mean ejection fraction (55.8 ± 7.58) [21]. The specific analysis of different phenotypes based on LVEF within the context of HF and T1D, remains insufficiently documented, presenting an area that warrants further research.

Regarding risk factors, in a study of 33,402 patients with T1D over a mean follow-up period of 7.9 years, Rosegren et al. found that, besides female gender, worse glycemic control and the presence of albuminuria were associated with an increased risk of HF. Interestingly, even well-controlled diabetes and normoalbuminuria were linked to an elevated risk of HF, though it was not as pronounced in those with both well-controlled diabetes and normoalbuminuria [18]. Furthermore, a Danish cohort of T1D subjects with either diastolic or systolic subclinical myocardial dysfunction, when compared to a control group, had a longer history of diabetes (35.1 ± 14.9 vs. 30.1 ± 15.5 years; *p* = 0.005), a higher body mass index (BMI) (26.1 ± 3.9 vs. 25.0 ± 3.7 kg/m^2^; *p* = 0.013), higher systolic blood pressure (143 vs. 136 mmHg; *p* < 0.001), and lower kidney function (eGFR 75.4 ± 26.2 vs. 83.7 ± 21.0 mL/min/1.73m^2^; *p* = 0.003). Additionally, they were more likely to be on statin (*p* = 0.039) and antihypertensive medications (*p* < 0.001), and showed a higher prevalence of advanced retinopathy and albuminuria stages (*p* < 0.001 for both comparisons) [21] (Table 2).

**Table 2:** Risk factors for developing heart failure in patients with type 1 diabetes

Non-modifiable risk factors	Female gender
	Age
	Longer duration of diabetes
	Myocardial infarction
	Chronic kidney disease
Potentially modifiable risk factors	Hypertension
	Poor glycemic control
	Increased body mass index
	Albuminuria
	Lipid profile
	Tobacco smoking

The DCCT/EDIC study revealed that glycemic control, measured by glycated hemoglobin (HbA1c) was the most significant modifiable risk factor for congestive HF in 1441 patients with T1D over 29 years. For every 1% increase in HbA1c, there was a 3.15-fold higher risk of HF. Early intensive therapy appeared to reduce the long-term risk of HF five fold compared to conventional treatment; however, the 30-year analysis included relatively few HF events, preventing a definitive conclusion [22]. In line with these results, Lind et al. found that patients with elevated HbA1c levels (≥ 10.5%) experienced a > tenfold increased risk of morbidity and mortality from CVD, particularly HF. This risk escalated with age and the duration of diabetes and was further exacerbated by modifiable factors such as smoking, high systolic blood pressure, and elevated BMI. Additionally, a history of acute myocardial infarction contributed to an increased risk of HF. On the other hand, higher levels of HDL cholesterol (HDL-c) were associated with a reduced risk of HF, while LDL cholesterol levels showed no significant correlation [23].

Moreover, in a study of 78 adolescents with a 6-year history of T1D, despite normal cholesterol and lipid levels, a significant number had microalbuminuria and diastolic dysfunction. Female patients with diastolic dysfunction had lower HDL-c levels (OR 0.93; 95% CI 0.88–0.99; *p* = 0.029) and higher total cholesterol (TC)/HDL-c (OR 2.55; 95% CI 1.9–5.45; *p* = 0.016) and triglyceride (TG)/HDL-c (OR 2.74; 95% CI 1.12–6.71; *p* = 0.028) ratios, which were linked to diastolic complications. The cutoff values for predicting diastolic dysfunction were 49 mg/dL for HDL, 3.0 for TC/HDL-c, and 1.85 for TG/HDL. These findings suggest that these ratios may help predict diastolic dysfunction in young female patients with poorly controlled T1D [24].

Regarding mortality, a UK study examined the impact of DM on mortality and the occurrence of HF, with a focus on gender differences. Results showed that individuals with DM had nearly twice the risk of mortality and HF compared to those without. Notably, women with DM, especially T1D, had a significantly higher risk of HF than men with DM, independent of other risk factors. This gender-diabetes interaction was more pronounced in T1D [25]. Furthermore, in a Swedish study involving 27,195 individuals with T1D and 135,178 controls with a median follow-up period of 10 years, 924 T1D patients and 1405 controls died. The findings showed that individuals who developed T1D between 0 and 10 years of age had significantly higher hazard ratios for various outcomes compared to controls, including a 4.11-fold risk of death, a 7.38-fold risk of cardiovascular death, an 11.44-fold risk of CVD, a 30.50-fold risk of coronary heart disease, a 30.95-fold risk of acute myocardial infarction, a 6.45-fold risk of stroke, a 12.90-fold risk of HF, and a 1.17-fold risk of atrial fibrillation. For those who developed T1D between the ages of 26 and 30, the risks were lower but still high. The overall incidence rate for all-cause mortality in T1D patients was 1.9 per 100,000 person-years. Developing T1D before 10 years of age resulted in a greater loss of life-years compared to diagnosis between 26 and 30 years of age, with women losing 17.7 and men losing 14.2 life-years in the former group and 10.1 and 9.4 life-years in the latter group, respectively. The study underscores the substantial impact of age at T1D onset on mortality and cardiovascular risks [26].

On diabetes onset, a recent study comparing Latent Autoimmune Diabetes in Adults (LADA) to T2D revealed similar risks of death (HR 1.44; 95% CI 1.03, 2.02 vs. 1.31,95% CI, 1.03, 1.67) and CVD, including HF (HR 1.22; 95% CI 0.82, 1.62 vs. 1.53, 95% CI, 1.17, 2.00). However, LADA individuals exhibited a higher risk of diabetic retinopathy and poorer glycemic control. Two LADA subgroups emerged based on autoantibody levels: lower GADA levels were more likely to have CVD at the time of diagnosis and linked to higher risks of recurrent CVD and mortality, while higher GADA levels were associated with poor glycemic control and increased risk of CVD after diagnosis [27]. The main result of this study aligns with the findings of the UKPDS and other research studies [28, 29].

Moreover, a cohort study that evaluated the significance of risk factors and previous CVD, HF, and chronic kidney disease (CKD) for mortality in 36,303 T1D patients, revealed that older age (> 60 years), male gender, high HbA1c (> 7.8%), high blood pressure, a history of CVD, albuminuria, and advanced CKD were all associated with an increased risk of death. Subjects with a combination of CKD, CVD, and HF, exhibited a markedly increased risk of dying prematurely. The highest mortality rates were seen in people with the lowest renal function (eGFR stages G4–G5), or with a history of CVD, but especially in those with a history of HF. This underscores the importance of managing risk factors and addressing cardiovascular and renal complications in people with T1D [30].

## Pathophysiology of diabetes-associated HF

The mechanisms responsible for the association between DM and HF are complex and not fully understood. It is known that the primary contributors to HF in patients with DM include coronary artery disease (CAD) as well as arterial hypertension. However, numerous experimental and clinical studies have reported a direct harmful impact of DM on the myocardium. The presence of myocardial dysfunction in the absence of overt clinical CAD, valvular disease, and other conventional cardiovascular risk factors such as hypertension has led to the use of the term diabetic cardiomyopathy (DCM) [10]. The existence of this specific form of cardiomyopathy was first proposed in 1972 after post-mortem studies [31], based on the discovery of HF in individuals with DM who showed no signs of detectable CAD. Further investigations subsequently yielded more conclusive evidence of DCM in diabetic subjects without CAD [32]. This entity is based on the concept that diabetes itself is the key factor that induces structural and/or functional changes leading to the development of progressive left ventricular (LV) dysfunction. However, the existence of a cardiomyopathy as a distinct clinical entity is still uncertain and continues to be a subject of controversy. Indeed, it is reasonable to expect that this form of cardiomyopathy may also be present in diabetics who have concomitant CAD and/or hypertension. Nevertheless, assessing the specific impact of DCM on overall ventricular dysfunction in such cases is a significant challenge.

On the other hand, DCM is frequently an unrecognized pathological process and the exact prevalence remains uncertain because the disease follows a subclinical and asymptomatic course during its initial stage. The presence of LV dysfunction in diabetic subjects is estimated to be around 15–20%, but diastolic dysfunction, an early functional alteration in the diabetic myocardium, can be detected in up to 25–60% using conventional and Doppler ultrasound [33]. Although the concept of DCM is often considered in subjects affected by T2D, a metabolically-induced cardiomyopathy is also evident in subjects with T1D. In T1D, the presence of diastolic dysfunction has been demonstrated even in adolescents and young adults, as a potential early marker of HF [18, 34, 35].

**Fig. 1:**
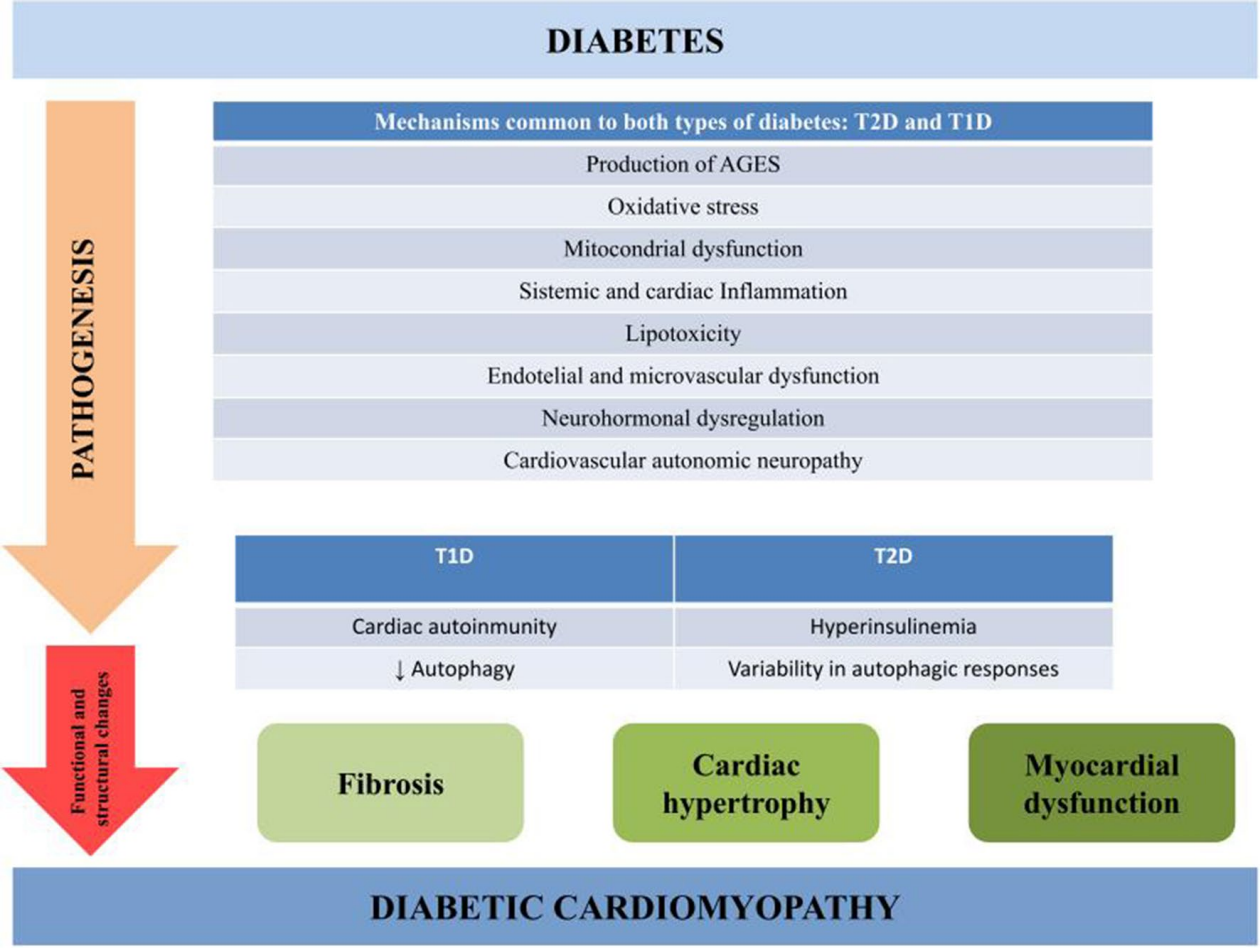
Potential mechanisms implicated in the pathophysiology of diabetic cardiomyopathy, and differential features in both types of diabetes. *AGE* Advanced glycation end products, *T2D*, Type 2 diabetes mellitus, *T1D* Type 1 diabetes mellitus

DCM is characterized by cardiac hypertrophy, interstitial fibrosis, cardiomyocyte apoptosis and associated diastolic and/or systolic myocardial dysfunction, and eventually by clinical HF [36–38]. Pathogenic mechanisms implicated in the development and progression of DCM are likely to be complex and multifactorial, from altered myocardial metabolism (hyperglycemia, hyperinsulinemia, lipotoxicity) to inflammation and oxidative stress, renin–angiotensin–aldosterone activation, microvascular dysfunction, cardiac autonomic neuropathy, or cardiac autoimmunity, among other things [39]. Most of these mechanisms are closely interrelated. Diabetic cardiomyopathy has been extensively studied in T2D, while its mechanisms in T1D are not fully understood. Although T1D and T2D differ in etiology and metabolic profiles, the two types share many features of cardiomyopathy [37]. However, specific mechanisms have been documented only in T1D. Figure 1 schematically represents the common and differential potential pathophysiological mechanisms involved in the onset and progression of DCM in both types of diabetes. Next, we will provide a concise overview of the main primary pathways associated with myocardial dysfunction, with a particular focus on findings related to T1D.

### Molecular and cellular mechanisms contributing to diabetes-associated HF in type 1 diabetes

#### Hyperglycemia and advanced glycation end products (AGEs)

One of the most well established mechanisms linking DM, including T1D, to HF is chronic hyperglycemia. Both preclinical and clinical evidence strongly suggests that hyperglycemia plays a causal role in diabetes-related HF, including in T1D [19, 23, 40, 41]. In experimental models of T1D diabetic cardiomyopathy, the improvement of hyperglycemia mitigates diabetes-associated diastolic dysfunction [42]. Chronic hyperglycemia results in the exacerbation of two potentially pathological molecular processes: non-enzymatic glycation with the formation of advanced glycation end products (AGEs) and oxidative stress, both intricately linked. AGEs may play a pivotal role in the development and progression of DCM by stimulating collagen expression and accumulation, contributing to myocardial fibrosis and stiffness, and diastolic dysfunction [43, 44].

#### Oxidative stress and mitochondrial dysfunction

Additionally, chronic hyperglycemia increases mitochondrial activity, promoting the production of reactive oxygen species (ROS) and elevated oxidative stress. These effects trigger an inflammatory process in the myocardium, leading to fibrosis and cardiac remodeling, disruption of calcium homeostasis, endothelial dysfunction, and ultimately a reduction in cardiac contractility and relaxation [36, 39]. In several mouse models of T1D, therapeutic targeting focused on oxidative stress was associated with suppressed high glucose-induced superoxide generation and enhanced mitochondrial function, with an effect in preventing cardiac remodeling and dysfunction in a setting of DM [45–47].

Mitochondrial dysfunction plays a pivotal role in DCM and is usually found in cardiac tissue in T1D [37, 48, 49]. Decreased mitochondrial oxidative capacity is caused by altered mitochondrial ultrastructure, proteomic remodeling, and oxidative damage to proteins and mitochondrial DNA [47, 50]. Additional mechanisms for mitochondrial dysfunction comprise perturbed mitochondrial Ca2+dynamics, mitochondrial uncoupling in T2D, and decreased cardiac insulin signaling in T1D [48, 49].

#### Inflammation

On the other hand, chronic inflammation plays a key role in the pathogenesis of HF in diabetes, especially in HF with preserved ejection fraction [49, 51–53]. It is well established that DM is a pro-inflammatory state [54]. This inflammatory milieu can cause direct damage to cardiac myocytes, leading to myocardial dysfunction. Additionally, inflammation contributes to the formation and progression of atherosclerosis, a key factor in HF development. Several systemic inflammatory biomarkers have been described as being associated with CVD, including HF [55]. In particular, in a study by Puig et al., the systemic pro-inflammatory molecule GlycA, a novel biomarker of protein glycan N‐acetyl groups, was associated with the presence of myocardial dysfunction in T1D subjects [21].

In relation to cardiac inflammation, studies using experimental models of diabetes have identified a critical role for increased myocardial inflammation in the progression of DCM [56]. Hearts from T1D mice and rats showed increased infiltration by leukocytes, such as macrophages, which raised levels of pro-inflammatory cytokines (TNFα, IL-1β, IL-6), increased the expression of vascular cell adhesion molecule-1 and intercellular adhesion molecule-1, and decreased the activity of the collagen-degrading matrix metalloproteinase (MMP), leading to profibrotic responses and cardiac remodeling [51, 57]. Therapies that target proinflammatory signaling have been shown to attenuate the development of experimental diabetic cardiomyopathy associated with a reduction in myocardial inflammation and cardiac fibrosis [56–58]. Nevertheless, clinical trials of anti-inflammatory and anti-cytokine therapies have shown limited cardioprotective benefits, in some cases even inducing adverse effects [52]. Moreover, studies in mouse models of T1D have detected higher T-cell infiltration in the myocardium, and certain efforts to mitigate cardiac fibrosis by reducing T-cell movement have proven effective [59, 60].

#### Lipotoxicity

Lipotoxicity and cardiac lipid accumulation in the heart have also been implicated in the development of DCM [61–63]. Studies on myocardial metabolism have demonstrated reduced glucose uptake and increased fatty acid (FA) uptake in individuals with T1D. In T1D, the deficiency of insulin promotes the release of FAs from adipose tissue, leading to a heightened presence of excess FAs in various tissues, including the myocardium. Under physiological conditions, the myocardium can utilize fatty acids and glucose as energy substrates, being able to switch energy sources depending on their relative availability, a condition known as metabolic flexibility. When an excessive amount of FAs exceeds the cell’s oxidative capacity, the FAs will accumulate, leading to a rise in metabolic stress and a significant reduction in cardiac efficiency and function. Additionally, the accumulation of FAs stimulates the production of intermediate products (ceramides, diacylglycerol, and ROS) which accumulate in the cardiomyocyte’s cytoplasm and lead to its apoptosis [64–66].

#### Endothelial and microvascular dysfunction

Microangiopathy has been shown to be present in the myocardium of diabetic patients. Autopsy samples of ventricular myocardium analyzed through traditional histological methods have revealed signs such as capillary basement membrane thickening, arteriole medial thickening, and perivascular fibrosis [37, 67, 68]. The possible mechanisms promoting microangiopathy in DCM are hyperglycemia, hyperlipidemia, and activation of the neurohormonal system. These factors may act either independently or synergistically, giving rise to oxidative stress, alterations in cellular signaling, and gene transcription. The microvascular changes result in reduced myocardial perfusion, subsequently compromising energy levels and leading to alterations in calcium handling, apoptosis, and diminished cardiac contractile strength [69].

Impaired endothelial function is a typical finding in DCM. In subjects with T1D hyperglycemia and oxidative stress impair endothelial function [70]. This endothelial dysfunction results in reduced bioavailability of nitric oxide, a molecule responsible for vasodilation and maintaining blood vessel health. With compromised endothelial function, there is an increased risk of hypertension and atherosclerosis, both of which are risk factors for HF. In the clinical settings, a link between coronary microvascular dysfunction and concurrent albuminuria has been reported. In T1D patients without a known history of heart disease, both microalbuminuria and macroalbuminuria have been associated with the presence of subclinical myocardial dysfunction [71].

#### Neurohormonal dysregulation and cardiovascular autonomic neuropathy

Activation of the renin–angiotensin–aldosterone system (RAAS) contributes to myocardial dysfunction [72–74]. Therefore, significantly more impaired cardiac sympathetic nervous system activity has been reported in HF patients with DM compared with HF patients without [75], and this is associated with adverse outcomes [76, 77]. Activation of the adrenergic system increases β-adrenergic expression and signaling, promoting myocyte hypertrophy, interstitial fibrosis, myocyte apoptosis, and contractile dysfunction [78]. In experimental models of T1D, an elevation in angiotensin-II receptor density and synthesis has been observed [57, 79]**.**

On the other hand, although cardiovascular autonomic neuropathy (CAN) is one of the least understood of all serious complications of diabetes, cardiac sympathetic signals play an important role in the perfusion of myocardial injury [80]. CAN is associated with imbalance between sympathetic and parasympathetic components of the autonomic nervous system. For instance, heightened cardiac sympathetic tone may lead to a decrease in myocardial vascularity, induce vascular hyperreactivity, heighten mitochondrial production of reactive oxygen species, disrupt intracellular signaling, trigger myocardial apoptosis, and encourage myocardial remodeling [39, 81]. Clinically it is associated with rest tachycardia, exercise intolerance, orthostatic hypotension and silent myocardial ischemia.

CAN is known to occur in individuals with T1D, correlating with increased CVD and mortality [79, 80]. It is suggested that cardiac neuropathy may affect up to 40% of individuals with T1D [82, 83]. However, CAN is more commonly associated with T2D, and it has been independently associated with LV diastolic dysfunction, even in asymptomatic T2D patients without any history of CVD [82]. In the study conducted by Maddaloni et al., it was observed that the prevalence of CAN is significantly higher in individuals with T2D compared to those with autoimmune diabetes (LADA and T1D) (64% vs. 40% vs. 26%; p < 0.001) [84]. Moreover, the study showed that individuals with LADA are 2.7 times less likely to develop CAN than those with T2D, even with a similar disease duration, irrespective of age and gender [84]. Conversely, after adjusting for pre-specified confounders and age, the risk of CAN in LADA was found to be similar to that in T1D. Long-standing diabetes and poor glycemic control are considered the main risk factors for the development of CAN in T1D [81, 85]. Strict glycemic control can prevent the development or delay the progression of CAN in subjects with T1D [86]. Some observational studies suggest that the presence of CAN is associated with the impairment of systolic and diastolic LV function [87].

#### Cardiac autoimmunity

A role for autoimmune mechanisms in the development of DCM is another point of recent interest. In observational studies, the presence of autoantibodies against heart muscle proteins is associated with subclinical myocardial dysfunction in subjects with T1D, independent of traditional CV risks. A study published by Sousa et al. involving 892 subjects with T1D being followed in the DCCT observed higher levels of cardiac autoantibodies in those who had inadequate glycemic control. Subjects who tested positive for two cardiac autoantibodies were more likely to have subclinical myocardial dysfunction and had an increased risk of higher cardiovascular disease. Using cardiac magnetic resonance indices, subjects with ≥ 2 autoantibodies were shown to have markedly greater LV end-diastolic volume (EDV), end-systolic volume (ESV), and LV mass, as well as a lower LVEF [88]. Chronic hyperglycemia causes myocardial damage and is associated with the release of myocardial proteins into the circulation. This could potentially result in the exposure of previously sequestered cardiac antigens, including α-myosin, to the immune system. Previous experimental studies have shown that the immune system is normally enriched in autoreactive CD4 + T cells specific for cardiac myosin due to loss of immunological tolerance [89].

#### Autophagy

A newly identified pathway in the development of DCM is the concept of autophagy [46]. Autophagy is a highly conserved cellular process that recycles long-lived proteins and organelles to uphold cellular equilibrium. Dysregulated autophagy has been linked to the pathogenesis of numerous ailments, including infectious diseases, cancer, obesity, and various cardiac conditions, such as DCM [90–92].

Several investigations have explored the potential connection between disrupted autophagy and the onset of DCM [91]. Within heart tissue, the elimination of damaged mitochondria through autophagy plays a vital role in preserving the well-being of cardiomyocytes. Damaged mitochondria resulting from cardiac injuries can generate ROS and release factors that induce cell death, thereby exacerbating cardiac harm. Nevertheless, excessive or prolonged autophagy can prove detrimental if it leads to cardiac atrophy [91]. Research findings in the context of DCM have yielded contradictory results. There is sufficient evidence from rodent model studies to indicate that cardiac autophagy is reduced in T1D [90, 93–95]. However, the functional consequence of this reduction in autophagy remains unclear. One suggested hypothesis is that impaired autophagy plays a role in causing cardiac damage by reducing the removal of dysfunctional organelles and protein aggregates. It is believed that enhancing autophagy could potentially mitigate damage in the hearts of subjects with T1D. On the contrary, Xu et al. have proposed that the reduced cardiac autophagy observed in T1D mice is actually an adaptive response aimed at preventing excessive autophagic degradation of cellular components [90]. However, autophagy may play a different role in T2D. Results from experimental T2D studies involving animals are less consistent, showing that cardiac autophagy can be either unchanged [96], reduced [97, 98], or even increased [99, 100]. Additional research is required to explore the underlying mechanisms responsible for the differences in autophagy observed in T1D compared to T2D.

### Diabetes-related comorbidities

T1D often coexists with other metabolic disorders, such as dyslipidemia and obesity. These comorbidities further increase the risk of HF. Dyslipidemia can lead to the development of atherosclerosis, while obesity contributes to insulin resistance and exacerbates hyperglycemia, augmenting the cardiovascular burden.

T1D patients show significant qualitative and functional abnormalities of lipoproteins that are likely to be implicated in the development of atherosclerosis and premature CVD. Subjects with T1D, particularly women with suboptimal glycemic control, exhibit an altered lipid profile characterized by elevated triglyceride levels and reduced HDL concentrations (HDL-c). Improving glycemic control has been shown to normalize most of these changes, with the exception of HDL-c [101]. In relation to lipoprotein quality, intensive diabetes therapy has been linked to potentially beneficial alterations in circulating LDL-c and HDL-c subclasses in T1D [102].

Relationship between advanced metabolic profile and atherosclerotic CVD in T1D has been reported [21]. On the other hand, the presence of diabetic dyslipidemia may also contribute to diabetic myocardial dysfunction. In particular because the excess flux of mobilized FAs to the liver promotes overproduction of TG-rich lipoproteins (TGRLs) and their remnants. Higher numbers of circulating TGRLs are frequently associated with increased concentrations of remnant cholesterol and with reduced HDL-c, and all contribute to the development of ischemic heart disease [103]. However, their contribution, if any, on non-ischemic cardiomyopathy remains poorly explored. In a recent study involving 1093 T1D subjects without known heart disease, TGRLs, such as VLDL (total VLDL particles, large VLDL subclass, and VLDL-TG content) and IDL were associated with the presence of subclinical myocardial dysfunction [21].

In summary, numerous mechanisms have been identified that can contribute to myocardial remodeling and LV dysfunction in DM, including T1D. Diabetic cardiomyopathy was initially described as a phenotype of dilated cardiomyopathy with systolic LV dysfunction [31]. However, in recent years, the presence of diastolic dysfunction is regarded as the first manifestation of DCM. Traditionally, two stages have been identified: an initial phase characterized by LV hypertrophy, increased myocardial stiffness, increased atrial filling pressure, and altered diastolic function (restrictive phenotype/HFpEF), and a later stage characterized by increased cardiac fibrosis, further deterioration in diastolic function, and the onset of systolic dysfunction (dilated phenotype/HFrEF) [104, 105]. Nevertheless, there is controversy regarding whether these two phenotypes are successive stages or instead independent phenotypes. The evaluation of myocardial dysfunction using more advanced techniques for assessing systolic/diastolic function in the preclinical stage of DCM has shown the presence of systolic dysfunction in the course of normal diastolic function. Employing these techniques, Seferovic et al., have recently found evidence favoring the notion of two independent clinical phenotypes rather than successive stages of the same disease [106]. Whereas both phenotypes are characterized by disparities in structural and functional aspects, they differ in their underlying pathophysiological mechanisms. In the restrictive phenotype, hyperglycemia, lipotoxicity, and insulin resistance are the primary mechanisms that induce left ventricular remodeling with myocardial and interstitial fibrosis. In the dilated phenotype, the loss of cardiomyocytes is a consequence of oxidative stress generated by microvascular damage and autoimmune-related inflammatory cells, with a possible role for hyperglycemia and lipotoxicity as well. Distinguishing between these two forms could have important prognostic and therapeutic implications.

## Screening and diagnosis of HF

**Fig. 2:**
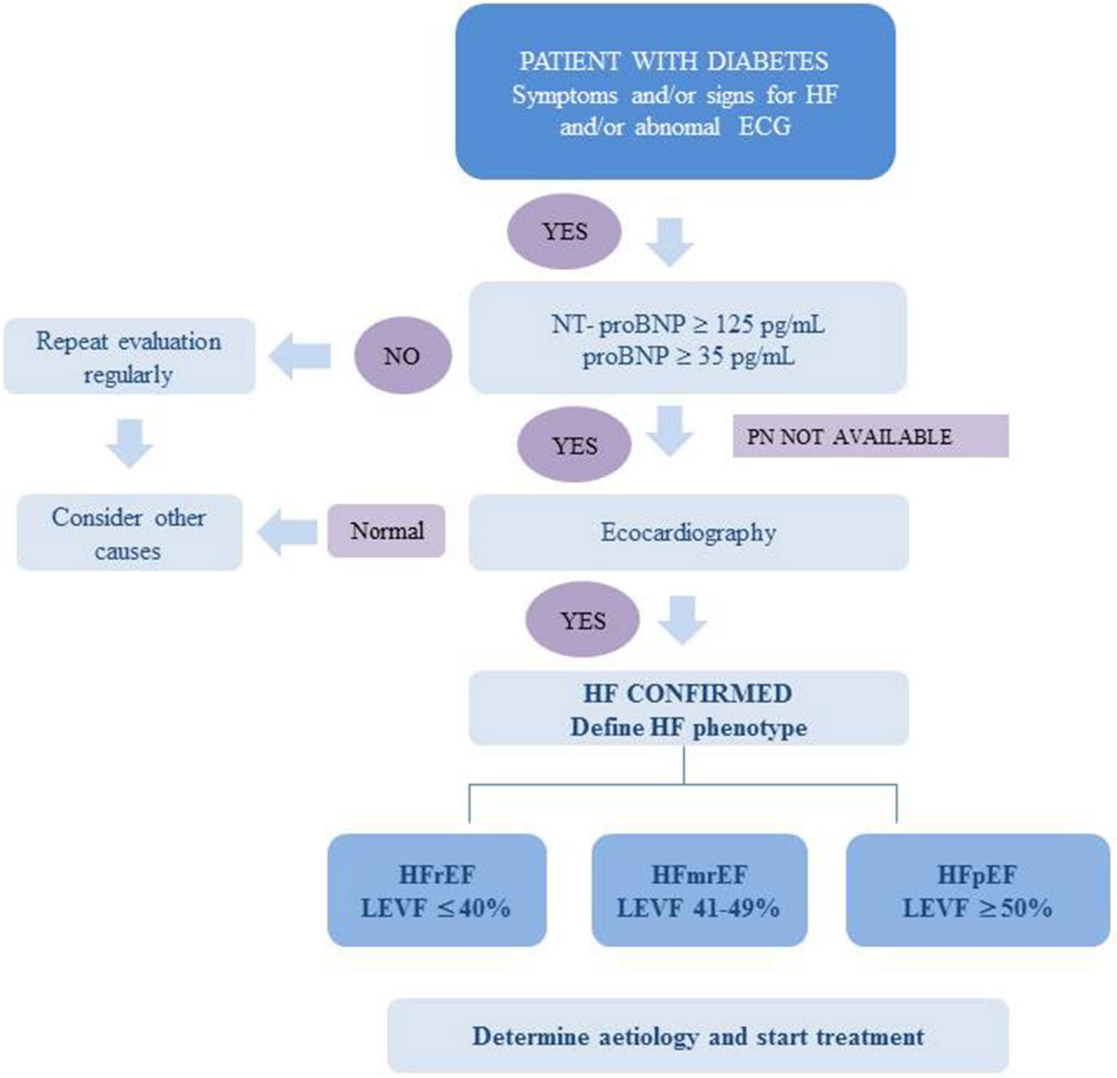
Diagnostic and screening algorithm for heart failure in individuals with diabetes. Adapted from the European Society of Cardiology Guidelines for the management of cardiovascular disease in diabetes 2023 [108]. *BNP* B-type natriuretic peptide, *ECG* electrocardiogram, *HF* heart failure, *HFmrEF* heart failure with mildly reduced ejection fraction, *HFpEF* heart failure with preserved ejection fraction, *HFrEF* heart failure with reduced ejection fraction, *LVEF* left ventricular ejection fraction; *NT-proBNP* N-terminal pro-B-type natriuretic peptide, *PN* natriuretic peptide

The diagnosis of HF requires the presence of symptoms and/or signs of HF and objective evidence of cardiac dysfunction [107]. According to the recent recommendations of the 2023 ESC Guidelines for the management of CVD in diabetes, in order to identify the shift from being at risk of HF to actually developing it, healthcare providers should routinely assess for HF symptoms in clinical practice [108]. There are no specific recommendations regarding the diagnosis and screening of HF in patients with T1D. If one or more of the symptoms or signs is present and/or the patient has an abnormal electrocardiogram, HF can be suspected, and the measurement of natriuretic peptides (NPs; BNP, B-type natriuretic peptide; NT-proBNP, N-terminal pro-B-type natriuretic peptide) is recommended. A value of NT-proBNP or BNP below the cut-off point has a high negative predictive value and indicates a low probability of HF. On the other hand, elevated levels of NPs support a diagnosis of HF, and echocardiography is then recommended to assess cardiac function and markers of diastolic dysfunction (Fig. 2) [107, 108].

Screening for HF is a priority in individuals with DM since, as we have noted, HF constitutes an early, highly prevalent, and often undiagnosed complication. As we have seen in this review, a non-negligible proportion of patients with DM, including adolescents or young adults with T1D, have subclinical diastolic dysfunction. Therefore, these individuals are at higher risk of developing symptomatic HF. The 2022 AHA/ACC/HFSA guidelines classify DM as a preclinical state of HF and recommend the periodic measurement of NPs, even in individuals who have not developed symptoms. The use of NPs to rule out HF in DM is well validated [109]. A recent study found that elevated NT-ProBNP levels were independently linked to HF in a cohort of 664 individuals with T1D [HR 1.7 (95% CI 1.1–2.4), *p* = 0.01] [110].

On the other hand, the best approach to the diagnosis of DCM is the detection of functional and structural changes in the LV and the exclusion of other heart diseases [37]. For diastolic dysfunction in young individuals with T1D, the general guidelines provided by the American Society of Echocardiography and the European Association of Cardiovascular Imaging recommend using various indices such as pulse Doppler transmitral inflow velocities (E and A waves), tissue Doppler early and late mitral annular diastolic velocities (e0 and a0), measuring atrial size, and evaluating pulmonary venous flow [111]. Recent advances in ultrasound techniques have allowed for the detection of subtle cardiac abnormalities that conventional methods may miss, such as ventricular deformation and desynchrony indices. Other techniques such as cardiac magnetic resonance can increase the detection of subclinical myocardial dysfunction [112]. In a recent study, Kaushik et al., identified preclinical ventricular dysfunction with echocardiographic abnormalities in individuals with T1D [113]. Specifically, they observed lower LV strain indices in children and adolescents with T1D compared to non-diabetic controls, even though these individuals did not display overt HF and had normal LVEFs. These myocardial abnormalities were found to be correlated with HbA1c levels. Although LV diastolic dysfunction is the earliest sign of HF in individuals with DM, recent research has highlighted the role of left atrial dysfunction as a contributing factor. A study by Ifuku et al. observed left atrial dysfunction, particularly left atrial phasic strain, in adolescents and young people with T1D but not in non-diabetic controls [35]. The authors suggest that this could serve as an early and sensitive marker of diastolic dysfunction in T1D. Therefore, identifying cardiac dysfunction in asymptomatic individuals with T1D may support the development of effective therapeutic approaches for diabetic cardiomyopathy. This could enhance treatment for these patients and ultimately improve their prognosis.

## Therapeutic strategies for type 1 diabetes-associated HF

The optimal management of HF involves utilizing pharmacological and device-based treatments but also implementing lifestyle changes. The current pharmacological treatment of HF is based on the use of drugs that have been demonstrated scientifically to reduce the risk of hospitalization for HF and cardiovascular mortality. It is important to note that, except in the case of SGLT2 inhibitors, clinical trials in HF have not been conducted exclusively in patients with DM, so the available evidence is derived from subanalyses of mixed populations. The occurrence of DM among study participants ranged from 20% to nearly 50%, with most of them having T2D. Overall, all pharmacological and device-based therapies available for HF were similarly effective, regardless of the presence of DM [114]. Current guidelines for the treatment of acute and chronic HF published by the ESC (2021), AHA/ACC/HFSA (2022) and, more recently, ESC Guidelines for the management of CVD in diabetes (2023), do not recommend specific treatment approaches for patients with DM and HF, and treatments vary depending on LVEF [107, 108, 115]. Pharmacological and non-pharmacological treatments for HF according to LVEF are summarized in Table 3. The main goals of medical treatment for patients with HF include preventing recurrent hospitalization due to worsening heart failure, reducing mortality, and improving the quality of life and functional capacity [107, 108, 116].

**Table 3:** Pharmacological and non-pharmacological treatment of heart failure in patients with diabetes

Non-pharmacological	• Cardio-healthy diet low in salt (< 3 g per day) • Regular physical exercise (combination of aerobic and muscle strength) • Smoking cessation and reduced alcohol consumption • Management of cardiovascular and non-cardiovascular comorbidities (hypertension, obesity, kidney disease, lipid disorders, etc.) • Cardiac rehabilitation for eligible patients • Avoiding medications that may lead to decompensating (NSAIDs, COX-2 inhibitors, etc.) • Vaccination (influenza, pneumonia, COVID-19, tetanus) • Monitoring of weight and blood pressure, preferably daily • Patient and/or caregiver education • Prevention of hypoglycemia

	HFrEF (≤ 40%)	HFmrEF (41–49%)	HFpEF (≥ 50%)
Pharmacological	ACEI/sacubitril/ valsartan Β-blockers MRAs SGLT2i*	ACEI/sacubitril/ valsartan Β-blockers MRAs SGLT2i*	Comorbidities treatment + SGLT2i*

^*^ No randomized clinical trials have evaluated the effect of treatment with SGLT2i in HF and DM1

*NSAIDs* Nonsteroidal Anti-Inflammatory Drugs, *Cox-2 inhibitors* cyclooxygenase-2, *HF* heart failure; *HFrEF* heart failure with reduced ejection fraction, *HFmrEF* heart failure with mildly reduced ejection fraction, *HFpEF* heart failure with preserved ejection fraction, *ACEI* angiotensin-converting enzyme inhibitors, *MRAs* mineralocorticoid receptor antagonists, *SGLT2i* sodium-glucose cotransporter 2 inhibitors

### Lifestyle interventions

Lifestyle changes play a crucial role in the management of heart failure and diabetes, and are listed in Table 3. Regular medical follow-up, preferably within multidisciplinary units, patient education, and active participation in disease self-management are key aspects for improving clinical outcomes and patients’ quality of life.

### Pharmacological treatment of HF

#### Type 2 diabetes mellitus

Pharmacological treatment is the cornerstone of HF management and should be implemented concurrently with other non-pharmacological interventions. Classically, therapies in HF focused on the renin–angiotensin and sympathetic nervous system. Regarding HFrEF, large well-designed randomized controlled clinical trials have shown that angiotensin-converting enzyme inhibitors (ACEI) [117], angiotensin II receptor blockers (ARBs) [118], β-blockers [119, 120], mineralocorticoid receptor antagonists (MRAs) [121, 122], and, more recently sacubitril/valsartan (a neprilysin inhibitor/ARBs) [123] and ivabradine [124] have all resulted in significant reductions in CV events in terms of mortality and hospitalizations.

A significant breakthrough in contemporary management of HF was the finding that treatment with SGLT2 inhibitors was associated with a lower risk of HF hospitalization in patients with T2D and CV disease or at high risk thereof. A meta-analysis of six CV and renal outcome trials of four SGLT2 inhibitors (empagliflozin [125], canagliflozin [126], dapagliflozin [127] and ertugliflozin [128]) in patients with T2D (EMPA-REG OUTCOME, CANVAS Programme, DECLARE-TIMI-58, CREDENCE, VERTIS CV) demonstrated a 32% reduction in HF hospitalization [129]. These results indicated a potential benefit of SGLT2 inhibitors in treating individuals with established HF, although it should be noted that HF-related outcomes were not the primary focus of the study. Taking into account these findings, recent randomized clinical trials (RCTs) have been conducted involving patients with HFrEF (DAPA-HF [130] and EMPEROR-Reduced trials [131]) and HFpEF (EMPEROR-Preserved and DELIVER trials [130, 132]), in which HF outcomes were the primary objective, and including patients both with and without DM (almost 50% had T2D). In these large trials, treatment with SGLT2 inhibitors in combination with optimal medical therapy (ACEI/ARNI, β-blockers, and MRAs) in patients with symptomatic chronic HF is associated with a reduction in the risk of hospitalization for HF and cardiovascular mortality, regardless of the presence of DM and across all LVEF. Furthermore, there have also been reported improvements in symptoms and quality of life among patients with HF. Recent trials with SGLT2 inhibitors have also shown benefits concerning HF-related hospitalization and CV mortality in subjects admitted to the hospital due to acute decompensated HF (SOLOIST-WHF trial: sotagliflozin and EMPULSE trial: empagliflozin) [133, 134]. This positive effect was also observed regardless of LVEF or the presence of DM. Thus, based on strong evidence, the SGLT2 inhibitors dapagliflozin, empagliflozin, and more recently sotagliflozin (currently approved for the treatment of HF in the United States but not in the European Union) are recommended as first line therapy in patients with T2D and HF to reduce CV death and HF hospitalization [108].

Another pharmacological group of interest in terms of cardioprotective effects is the glucagon-like peptide 1 agonists (GLP1-RAs). Despite positive outcomes in reducing major cardiovascular events, studies have shown most GLP-RAs having a neutral effect on the risk of HF hospitalization in patients with T2D who had, or were at high risk of, CVD [135, 136]. Future studies are needed to investigate the effects of GLP1-RAs in HF and T2D as primary outcomes and as well as its benefits in certain populations such as non-diabetic or T1D subjects. Recently, treatment with a GLP1-RA (semaglutide) was associated with improved symptoms and exercise capacity in patients with HFpEF and obesity [137]. Moreover, in patients with preexisting cardiovascular disease and overweight or obesity, treatment with semaglutide resulted in a 20% reduction in the risk of a composite of death from cardiovascular causes, nonfatal myocardial infarction, or nonfatal stroke (HR 0.80; 95% CI, 0.72 to 0.90). Noteworthy, an 18% reduction for the HF composite endpoint (HR 0.82; 95% CI, 0.71 to 0.96) and a 21% reduction in hospitalization or urgent medical visit for HF (HR 0.79; 95% CI, 0.60 to 1.03) were observed [138].

#### Type 1 diabetes mellitus

In relation to T1D, it is worth noting that most large-scale trials involving medications (ACEI, ARBS, β-blockers, MRAs and sacubitril/valsartan) and medical devices for HF have had limited participation from individuals with T1D, often excluding them or lacking detailed information about this specific subgroup. As a result, the choice of treatment for individuals with T1D is primarily extrapolation from results observed in individuals with T2D. Thus, though the therapies employed for preventing and managing HF in T1D are similar, there is no strong evidence to support this approach [4, 114].

Moreover, it is important to note that in all of the large RCTs with SGLT2 inhibitors, patients with HF and T1D were consistently excluded. To our knowledge, there are no studies that have assessed the effects of SGLT2 inhibitor treatment in patients with T1D and HF, resulting in a lack of evidence and specific recommendations for this subgroup. In experimental models of T1D, treatment with dapagliflozin prevents intimal thickening, cardiac inflammation, and fibrosis [139]. Regarding glycemic control, several clinical trials have evaluated the efficacy and safety of the use of SGLT2 inhibitors in T1D [140–143]. Treatment with SGLT2 inhibitors added to adjunctive therapy with basal-bolus regimen insulin have demonstrated reduced HbA1c and lower glucose variability with increased time in optimal glucose range as well as additional benefits in terms of reductions in weight and insulin dose without increasing the incidence of hypoglycemia. Based on these positive results, dapagliflozin was the first SGLT2 inhibitor to have its marketing authorization extended to T1D with a BMI ≥ 27 kg/m^2^. However, ‘euglycemic ketoacidosis’ has been reported in 2–3% of patients with T1D taking SGLT2 inhibitors. The careful selection of individuals with T1D for SGLT2 inhibitor treatment is crucial for minimizing the risk of diabetic ketoacidosis (DKA). This treatment may be considered for subjects between the ages of 18 and 74 who are overweight or obese, have been on stable and optimized insulin therapy (not recently diagnosed), require a high dose of insulin (i.e., > 0.5 units/kg per day), presentation with ketone levels < 0.6 nmol/L, and have demonstrated adherence to their insulin regimen as well as the ability to understand and apply relevant education regarding the risk of DKA [140]. In our opinion, when weighing the use of SGLT2 inhibitors in T1D for the treatment of asymptomatic HF, it is essential to establish strategies to reduce the risk of DKA, ideally with the involvement of specialized multidisciplinary units. This entails providing comprehensive education to both individuals with T1D and healthcare professionals about the potential risk of DKA and, if it arises, the methods by which it can be mitigated. It is crucial to closely monitor ketone levels and consider recommendations for temporary suspension in specific circumstances (such as during fasting, vigorous physical activity, concurrent medical illness, recurrent vomiting, alcoholism, etc.).

### Stage-based treatment of HF

According to the severity of symptoms and the presence of structural heart disease, the ACC/AHA/HFSA classified HF into four distinct stages. Stage A includes individuals at high risk of developing HF, such as patients with diabetes, and focuses on preventive measures, including lifestyle changes and management of risk factors. Stage B targets patients with structural heart disease but no symptoms, utilizing medications such as ACEI, ARBs and β-blockers to delay the onset of HF symptoms. In Stage C, for patients with structural disease and symptoms, medications include diuretics, ACEI or ARBs, -blockers, MRAs, sacubitril/valsartan, ivabradine, implantable cardioverter-defibrillators, and cardiac resynchronization therapy-defibrillators, to manage symptoms and improve quality of life. Stage D, the most advanced stage, focuses on managing symptoms and prolonging life in patients with refractory HF, utilizing specialized interventions such as mechanical circulatory support devices, and, in some cases, heart transplantation. For patients with diabetes, SGLT2 inhibitors are recommended from stage B, but thiazolidinediones and DPP4i saxagliptin, should be avoided due to the increased risk of HF admission linked to their use [117, 144].

### Glycemic control

In addition to the monitoring of blood pressure and body weight as well as lipid control, a holistic approach to HF management in subjects with DM should also include glycemic control. The effect of chronic hyperglycemia on micro and macrovascular complications has been firmly established in longitudinal studies involving both subjects with T2D and subjects with T1D [145–147]. It is also known that reducing HbA1c decreases microvascular complications [148]. However, the influence of glycemic optimization on the risk of cardiovascular events is more complex, and its impact in HF has not been clearly established. In T2D, more intensive glycemic control reduces the risk of microvascular disease but has not been proven to reduce the risk of macrovascular complications [149–152]. A meta-analysis that included 8 randomized trials (37,229 subjects) showed that more intensive glycemic control in patients with T2D did not reduce the occurrence of HF events [153]. Moreover, findings regarding optimization of glycemic control and its effects on diastolic dysfunction in patients with T2D have been conflicting [154, 155]. A large prospective study to assess long-term LVEF trajectory (up to 15 years) in T2D and HD did not find a significant relationship between the degree of glycemic control and recurrent HF admissions [156]. In contrast to what occurs in T2D, achieving near-normal HbA1c levels has demonstrated long-term beneficial effects on the incidence of CVD in T1D [22, 147, 149]. In the 30-year follow-up of the DCCT/EDIC trial, intensive glucose control led to a 30% reduction in the incidence of overall CVD, including CV death. Although HF was uncommon in this trial, the group that received intensive treatment showed a notable long-term reduction in the risk of HF.

The usual treatment for T1D is basal-bolus insulin therapy, and, as demonstrated, early intensive therapy seems to be crucial for reducing the long-term risk of HF. However, intensive diabetes therapy is associated with an increased risk of hypoglycemia. This adverse effect continues to be a significant challenge for subjects with T1D throughout their life span [147]. On the other hand, it is well established that hypoglycemia is associated with an increased risk of vascular events, especially in those with high CV risk. Evidence linking hypoglycemia to CVD comes predominantly from studies involving T2D patients. Severe hypoglycemia was associated with higher HF hospitalization in most of these studies. Although severe hypoglycemic events were associated with higher HF hospitalization [157–160], recent analyses have revealed a bi-directional association between hypoglycemia and CV outcomes, including HF. This suggests that causality is not straightforward, and hypoglycemia may be indicative of underlying frailty, or vice versa [157, 158]. Several observational studies have found a U-shaped relationship between HbA1c and all-cause mortality in patients with T2D and chronic HF. Consequently, patients with either very low or very high HbA1c levels were at a higher risk [161]. The lowest risk was found in those with modest glycemic control (HbA1c 7.1–8.0%) [162]. In T1D, despite the even greater risk of hypoglycemia, very few studies have investigated whether hypoglycemia may also increase the risk of CVD or death in this population. In most studies, severe hypoglycemic events have been associated with an increased risk of CVD and all-cause mortality, but data regarding HFoutcomes has not usually been reported [163–167].

In addition to hypoglycemia, glycemic variability (GV), measured as glucose oscillations intra- and interday, is emerging as an independent risk factor and predictor of worse CV outcomes. Recent clinical data indicate that GV is associated with increased risk of hypoglycemia, microvascular and macrovascular complications, and mortality in patients with DM, independently of HbA1c level [168–171]. Interestingly, greater GV has been observed in individuals with T1D compared to those with T2D. While some studies have associated GV with the risk of CAN in T1D, the substantial heterogeneity in the methodologies employed across various studies hinders any assertion of a causal relationship [172]. Experimental studies suggest that GV may contribute to CV complications through mechanisms such as oxidative stress, increased [170, 173]. Nevertheless, there remains a lack of substantial evidence supporting the beneficial impact of treating high GV to improve CV outcomes.

The technology applied to T1D has advanced significantly in recent years. Improvements in technological devices for diabetes management, such as continuous and intermittent glucose monitoring and hybrid closed-loop systems have improved glycemic control and resulted in overall decreases in the rates of hypoglycemia and as well as improved GV [116, 174]. Thus, device use may be associated with long-term prevention of T1D complications. However, there is still limited research on the direct effects of these devices on chronic complications in T1D [175]. Longitudinal studies indicate that using insulin pumps may help offset CV risk factors like hypertension and dyslipidemia [176, 177]. Additionally, pump users have been shown to have less arterial stiffness and better myocardial function. Data derived from registries and case–control studies have established an association between insulin pump use and a decreased incidence of CV events, including HF, and overall mortality rates [177, 178].

## Conclusion

Individuals with T1D face a significantly elevated risk of HF compared to those without DM. Despite the clear association between T1D and HF, the exact mechanisms are still not fully understood. Studies are needed to elucidate the underlying processes, pinpoint specific risk factors, and establish precise diagnostic biomarkers. On the other hand, evaluating comprehensive cardioprotection strategies and exploring adjunctive therapies are crucial. While certain therapeutic groups, such as SGLT2 inhibitors in T2D, show promise, their effectiveness and safety in T1D patients with HF remain uncertain and require further investigation.

## Data Availability

Not applicable. No new datasets were generated for this review article.

## Abbreviations

ACEI: Angiotensin-converting enzyme inhibitors
AGEs: Advanced glycation end products
AHA/ACC/HFSA: American Heart Association/American College of Cardiology/Heart Failure Society of America
BMI: Body mass index
CAD: Coronary artery disease
CAN: Cardiovascular autonomic neuropathy
CVD: Cardiovascular disease
DCCT: Diabetes Control and Complications Trial
DCM: Diabetic cardiomyopathy
DKA: Diabetic ketoacidosis
DM: Diabetes mellitus
eGFR: Estimated glomerular filtration rate
EDV: End-diastolic volume
ESV: End-systolic volume
ESC: European society of cardiology
FA: Fatty acid
GC: Glycemic variability
GLP1-RAs: Glucagon-like peptides 1 agonists
HbA1c: Glycated hemoglobin
HF: Heart failure
HFrEF: Heart failure with reduced ejection fraction
HFmrEF: Heart failure with mildly reduced ejection fraction
HFpEF: Heart failure with preserved ejection fraction
HDL: High-density lipoprotein
IR: Incidence rate
LDL: Low-density lipoprotein
LV: Left ventricular
LVEF: Left ventricular ejection fraction
MMP: Matrix metalloproteinase
MRAs: Mineralocorticoid receptor antagonists
NT-proBNP: N-terminal pro-B-type natriuretic peptide
NP: Natriuretic peptides
RAAS: Renin–angiotensin–aldosterone system
RCT: Randomized clinical trials
SGLT2: Sodium-glucose cotransporter 2 inhibitors
T2D: Type 2 diabetes
T1D: Type 1 diabetes
TC: Total cholesterol
TG: Triglycerides
CKD: Chronic kidney disease
TGRLs: TG-rich lipoproteins

## References

[1] Savarese G, Lund LH (2017). Global public health burden of heart failure. Card Fail Rev.

[2] Mosterd A, Hoes AW (2007). Clinical epidemiology of heart failure. Heart.

[3] Kannel WB, Hjortland M, Castelli WP (1974). Role of diabetes in congestive heart failure: The Framingham study. Am J Cardiol.

[4] Shaw JA, Cooper ME (2020). Contemporary management of heart failure in patients with diabetes. Diabetes Care.

[5] Packer M (2018). Heart failure: the most important, preventable, and treatable cardiovascular complication of type 2 diabetes. Diabetes Care.

[6] Conrad N, Judge A, Tran J, Mohseni H, Hedgecott D, Crespillo AP (2018). Temporal trends and patterns in heart failure incidence: a population-based study of 4 million individuals. Lancet.

[7] Dauriz M, Mantovani A, Bonapace S, Verlato G, Zoppini G, Bonora E (2017). Prognostic impact of diabetes on long-term survival outcomes in patients with heart failure: a meta-analysis. Diabetes Care.

[8] MacDonald MR, Petrie MC, Varyani F, Östergren J, Michelson EL, Young JB (2008). Impact of diabetes on outcomes in patients with low and preserved ejection fraction heart failure: an analysis of the candesartan in heart failure: assessment of reduction in mortality and morbidity (CHARM) programme. Eur Heart J.

[9] Ritchie RH, Abel ED (2020). Basic mechanisms of diabetic heart disease. Circ Res.

[10] Alonso N, Moliner P, Mauricio D (2018). Pathogenesis, clinical features and treatment of diabetic cardiomyopathy. Adv Exp Med Biol.

[11] Zannad F, Ferreira JP, Pocock SJ, Anker SD, Butler J, Filippatos G (2020). SGLT2 inhibitors in patients with heart failure with reduced ejection fraction: a meta-analysis of the EMPEROR-reduced and DAPA-HF trials. Lancet.

[12] De Ferranti SD, De Boer IH, Fonseca V, Fox CS, Golden SH, Lavie CJ (2014). Type 1 diabetes mellitus and cardiovascular disease: a scientific statement from the american heart association and american diabetes association. Diabetes Care.

[13] Roth GA, Forouzanfar MH, Moran AE, Barber R, Nguyen G, Feigin VL (2015). Demographic and epidemiologic drivers of global cardiovascular mortality. N Engl J Med.

[14] Van Riet EES, Hoes AW, Wagenaar KP, Limburg A, Landman MAJ, Rutten FH (2016). Epidemiology of heart failure: the prevalence of heart failure and ventricular dysfunction in older adults over time. a systematic review. Eur J Heart Fail.

[15] Avogaro A, Azzolina D, Fadini GP, Baldi I (2021). Incidence of heart failure in patients with type 1 diabetes: a systematic review of observational studies. J Endocrinol Invest.

[16] Haji M, Erqou S, Fonarow GC, Echouffo-Tcheugui JB (2023). Type 1 diabetes and risk of heart failure: a systematic review and meta-analysis. Diabetes Res Clin Pract.

[17] McAllister DA, Read SH, Kerssens J, Livingstone S, McGurnaghan S, Jhund P (2018). Incidence of hospitalization for heart failure and case-fatality among 3.25 million people with and without diabetes mellitus. Circulation.

[18] Rosengren A, Vestberg D, Svensson AM, Kosiborod M, Clements M, Rawshani A (2015). Long–term excess risk of heart failure in people with type 1 diabetes: a prospective case-control study. Lancet Diabetes Endocrinol.

[19] Cai X, Li J, Cai W, Chen C, Ma J, Xie Z (2021). Meta-analysis of type 1 diabetes mellitus and risk of cardiovascular disease. J Diabet Complicat.

[20] Konduracka E, Cieslik G, Galicka-Latala D, Rostoff P, Pietrucha A, Latacz P (2013). Myocardial dysfunction and chronic heart failure in patients with long-lasting type 1 diabetes: a 7-year prospective cohort study. Acta Diabetol.

[21] Puig-Jové C, Julve J, Castelblanco E, Julián MT, Amigó N, Andersen HU (2022). The novel inflammatory biomarker GlycA and triglyceride-rich lipoproteins are associated with the presence of subclinical myocardial dysfunction in subjects with type 1 diabetes mellitus. Cardiovasc Diabetol.

[22] Gubitosi-Klug RA, Lachin JM, Backlund JYC, Lorenzi GM, Brillon DJ, Orchard TJ (2016). Intensive diabetes treatment and cardiovascular outcomes in Type 1 diabetes: the DCCT/EDIC study 30-year follow-up. Diabetes Care.

[23] Lind M, Bounias I, Olsson M, Gudbjörnsdottir S, Svensson AM, Rosengren A (2011). Glycaemic control and incidence of heart failure in 20,985 patients with type 1 diabetes: an observational study. Lancet.

[24] Khedr D, Hafez M, Lumpuy-Castillo J, Emam S, Abdel-Massih A, Elmougy F (2020). Lipid biomarkers as predictors of diastolic dysfunction in diabetes with poor glycemic control. Int J Mol Sci.

[25] Chadalavada S, Jensen MT, Aung N, Cooper J, Lekadir K, Munroe PB (2021). Women with diabetes are at increased relative risk of heart failure compared to men: insights from UK biobank. Front Cardiovasc Med.

[26] Rawshani A, Sattar N, Franzén S, Rawshani A, Hattersley AT, Svensson AM (2018). Excess mortality and cardiovascular disease in young adults with type 1 diabetes in relation to age at onset: a nationwide, register-based cohort study. Lancet.

[27] Wei Y, Herzog K, Ahlqvist E, Andersson T, Nystrom T, Zhan Y (2023). All-cause mortality and cardiovascular and microvascular diseases in latent autoimmune diabetes in adults. Diabetes Care.

[28] Maddaloni E, Coleman RL, Pozzilli P, Holman RR (2019). Long-term risk of cardiovascular disease in individuals with latent autoimmune diabetes in adults (UKPDS 85). Diabetes Obes Metab.

[29] Luk AOY, Lau ESH, Lim C, Kong APS, Chow E, Ma RCW (2019). Diabetes-related complications and mortality in patients with young-onset latent autoimmune diabetes: a 14-year analysis of the prospective Hong Kong diabetes register. Diabetes Care.

[30] Eliasson B, Lyngfelt L, Strömblad SO, Franzén S, Eeg-Olofsson K (2022). The significance of chronic kidney disease, heart failure and cardiovascular disease for mortality in type 1 diabetes: nationwide observational study. Sci Rep.

[31] Rubler S, Dlugash J, Yuceoglu YZ, Kumral T, Branwood AW, Grishman A (1972). New type of cardiomyopathy associated with diabetic glomerulosclerosis. Am J Cardiol.

[32] Tj R, Mm L, Ss A, Ge L, Ha O, Mr A (1977). Evidence for cardiomyopathy in familial diabetes mellitus. J Clin Invest.

[33] Boyer JK, Thanigaraj S, Schechtman KB, Pérez JE (2004). Prevalence of ventricular diastolic dysfunction in asymptomatic, normotensive patients with diabetes mellitus. Am J Cardiol.

[34] Gøtzsche O, Darwish A, Gøtzsche L, Hansen L, Sørensen K (1996). Incipient cardiomyopathy in young insulin-dependent diabetic patients: a seven-year prospective doppler echocardiographic study. Diabet Med.

[35] Ifuku M, Takahashi K, Hosono Y, Iso T, Ishikawa A, Haruna H (2021). Left atrial dysfunction and stiffness in pediatric and adult patients with type 1 diabetes mellitus assessed with speckle tracking echocardiography. Pediatr Diabet.

[36] Huynh K, Bernardo BC, McMullen JR, Ritchie RH (2014). Diabetic cardiomyopathy: mechanisms and new treatment strategies targeting antioxidant signaling pathways. Pharmacol Ther.

[37] Miki T, Yuda S, Kouzu H, Miura T (2013). Diabetic cardiomyopathy: pathophysiology and clinical features. Heart Fail Rev.

[38] Jia G, Hill MA, Sowers JR (2018). Diabetic cardiomyopathy: an update of mechanisms contributing to this clinical entity. Circ Res.

[39] Marwick TH, Ritchie R, Shaw JE, Kaye D (2018). Implications of underlying mechanisms for the recognition and management of diabetic cardiomyopathy. J Am Coll Cardiol.

[40] Iribarren C, Karter AJ, Go AS, Ferrara A, Liu JY, Sidney S (2001). Glycemic control and heart failure among adult patients with diabetes. Circulation.

[41] Erqou S, Lee CTC, Suffoletto M, Echouffo-Tcheugui JB, De Boer RA, Van Melle JP (2013). Association between glycated haemoglobin and the risk of congestive heart failure in diabetes mellitus: systematic review and meta-analysis. Eur J Heart Fail.

[42] Tate M, Deo M, Cao AH, Hood SG, Huynh K, Kiriazis H (2017). Insulin replacement limits progression of diabetic cardiomyopathy in the low-dose streptozotocin-induced diabetic rat. Diab Vasc Dis Res.

[43] Ahmed N (2005). Advanced glycation endproducts–role in pathology of diabetic complications. Diabetes Res Clin Pract.

[44] Singh VP, Bali A, Singh N, Jaggi AS (2014). Advanced glycation end products and diabetic complications. Korean J Physiol Pharmacol.

[45] Ritchie RH, Love JE, Huynh K, Bernardo BC, Henstridge DC, Kiriazis H (2012). Enhanced phosphoinositide 3-kinase(p110α) activity prevents diabetes-induced cardiomyopathy and superoxide generation in a mouse model of diabetes. Diabetologia.

[46] De Blasio MJ, Huynh K, Qin C, Rosli S, Kiriazis H, Ayer A (2015). Therapeutic targeting of oxidative stress with coenzyme Q10 counteracts exaggerated diabetic cardiomyopathy in a mouse model of diabetes with diminished PI3K(p110α) signaling. Free Radic Biol Med.

[47] Huynh K, Kiriazis H, Du XJ, Love JE, Gray SP, Jandeleit-Dahm KA (2013). Targeting the upregulation of reactive oxygen species subsequent to hyperglycemia prevents type 1 diabetic cardiomyopathy in mice. Free Radic Biol Med.

[48] Bugger H, Abel ED (2010). Mitochondria in the diabetic heart. Cardiovasc Res.

[49] Riehle C, Bauersachs J (2019). Of mice and men: models and mechanisms of diabetic cardiomyopathy. Basic Res Cardiol.

[50] Wallace DC (1992). Mitochondrial genetics: a paradigm for aging and degenerative diseases?. Science.

[51] Riehle C, Bauersachs J (2019). Key inflammatory mechanisms underlying heart failure. Herz.

[52] Murphy SP, Kakkar R, McCarthy CP, Januzzi JL (2020). Inflammation in heart failure: JACC state-of-the-art review. J Am Coll Cardiol.

[53] Paulus WJ, Zile MR (2021). From systemic inflammation to myocardial fibrosis: the heart failure with preserved ejection fraction paradigm revisited. Circ Res.

[54] Diamant M, Lamb HJ, Smit JWA, De Roos A, Heine RJ (2005). Diabetic cardiomyopathy in uncomplicated type 2 diabetes is associated with the metabolic syndrome and systemic inflammation. Diabetologia.

[55] Akinkuolie AO, Buring JE, Ridker PM, Mora S (2014). A novel protein glycan biomarker and future cardiovascular disease events. J Am Heart Assoc.

[56] Jadhav A, Tiwari S, Lee P, Ndisang JF (2013). The heme oxygenase system selectively enhances the anti-inflammatory macrophage-m2 phenotype, reduces pericardial adiposity, and ameliorated cardiac injury in diabetic cardiomyopathy in zucker diabetic fatty rats. J Pharmacol Exp Ther.

[57] Westermann D, Rutschow S, Jäger S, Linderer A, Anker S, Riad A (2007). Contributions of inflammation and cardiac matrix metalloproteinase activity to cardiac failure in diabetic cardiomyopathy: the role of angiotensin type 1 receptor antagonism. Diabetes.

[58] Tschöpe C, Walther T, Escher F, Spillmann F, Du J, Altmann C (2005). Transgenic activation of the kallikrein-kinin system inhibits intramyocardial inflammation, endothelial dysfunction and oxidative stress in experimental diabetic cardiomyopathy. FASEB J.

[59] Lin Y, Tang Y, Wang F (2016). The protective effect of HIF-1α in T lymphocytes on cardiac damage in diabetic mice. Ann Clin Lab Sci.

[60] Abdullah CS, Li Z, Wang X, Jin ZQ (2016). Depletion of T lymphocytes ameliorates cardiac fibrosis in streptozotocin-induced diabetic cardiomyopathy. Int Immunopharmacol.

[61] Van De Weijer T, Schrauwen-Hinderling VB, Schrauwen P (2011). Lipotoxicity in type 2 diabetic cardiomyopathy. Cardiovasc Res.

[62] Ussher JR (2014). The role of cardiac lipotoxicity in the pathogenesis of diabetic cardiomyopathy. Expert Rev Cardiovasc Ther.

[63] Bayeva M, Sawicki KT, Ardehali H (2013). Taking diabetes to heart—deregulation of myocardial lipid metabolism in diabetic cardiomyopathy. J Am Heart Assoc.

[64] Ritchie RH, Zerenturk EJ, Prakoso D, Calkin AC (2017). Lipid metabolism and its implications for type 1 diabetes-associated cardiomyopathy. J Mol Endocrinol.

[65] Herrero P, Peterson LR, McGill JB, Matthew S, Lesniak D, Dence C (2006). Increased myocardial fatty acid metabolism in patients with type 1 diabetes mellitus. J Am Coll Cardiol.

[66] Diarte-Añazco EMG, Méndez-Lara KA, Pérez A, Alonso N, Blanco-Vaca F, Julve J (2019). Novel insights into the role of HDL-associated sphingosine-1-phosphate in cardiometabolic diseases. Int J Mol Sci.

[67] Kawaguchi M, Techigawara M, Ishihata T, Asakura T, Saito F, Maehara K (1997). A comparison of ultrastructural changes on endomyocardial biopsy specimens obtained from patients with diabetes mellitus with and without hypertension. Heart Vessel.

[68] Factor SM, Okun EM, Minase T (1980). Capillary microaneurysms in the human diabetic heart. N Engl J Med.

[69] Adameova A, Dhalla NS (2014). Role of microangiopathy in diabetic cardiomyopathy. Heart Fail Rev.

[70] Llauradó G, Ceperuelo-Mallafré V, Vilardell C, Simó R, Albert L, Berlanga E (2013). Impaired endothelial function is not associated with arterial stiffness in adults with type 1 diabetes. Diabet Metab.

[71] Jensen MT, Sogaard P, Andersen HU, Bech J, Hansen TF, Galatius S (2014). Prevalence of systolic and diastolic dysfunction in patients with type 1 diabetes without known heart disease: the thousand & 1 study. Diabetologia.

[72] Khatter JC, Sadri P, Zhang M, Hoeschen RJ (1996). Myocardial angiotensin II (Ang II) receptors in diabetic rats. Ann N Y Acad Sci.

[73] Sechi LA, Griffin CA, Schambelan M (1994). The cardiac renin–angiotensin system in STZ-induced diabetes. Diabetes.

[74] Ka C, Aj B, Dj K (2007). Angiotensin II and the cardiac complications of diabetes mellitus. Curr Pharm Des.

[75] Paolillo S, Rengo G, Pagano G, Pellegrino T, Savarese G, Femminella GD (2013). Impact of diabetes on cardiac sympathetic innervation in patients with heart failure: a 123I meta-iodobenzylguanidine (123I MIBG) scintigraphic study. Diabet Care.

[76] Gargiulo P, Acampa W, Asile G, Abbate V, Nardi E, Marzano F (2023). 123I-MIBG imaging in heart failure: impact of comorbidities on cardiac sympathetic innervation. Eur J Nucl Med Mol Imaging.

[77] Jacobson AF, Senior R, Cerqueira MD, Wong ND, Thomas GS, Lopez VA (2010). Myocardial iodine-123 meta-iodobenzylguanidine imaging and cardiac events in heart failure. results of the prospective ADMIRE-HF (adreview myocardial imaging for risk evaluation in heart failure) study. J Am Coll Cardiol.

[78] Falcão-Pires I, Leite-Moreira AF (2012). Diabetic cardiomyopathy: understanding the molecular and cellular basis to progress in diagnosis and treatment. Heart Fail Rev.

[79] Singh VP, Le B, Khode R, Baker KM, Kumar R (2008). Intracellular angiotensin II production in diabetic rats is correlated with cardiomyocyte apoptosis, oxidative stress, and cardiac fibrosis. Diabetes.

[80] Di Carli MF, Bianco-Batlles D, Landa ME, Kazmers A, Groehn H, Muzik O (1999). Effects of autonomic neuropathy on coronary blood flow in patients with diabetes mellitus. Circulation.

[81] Torry RJ, Connell PM, O’Brien DM, Chilian WM, Tomanek RJ (1991). Sympathectomy stimulates capillary but not precapillary growth in hypertrophic hearts. Am J physiol.

[82] Voulgari C, Psallas M, Kokkinos A, Argiana V, Katsilambros N, Tentolouris N (2011). The association between cardiac autonomic neuropathy with metabolic and other factors in subjects with type 1 and type 2 diabetes. J Diabet Complicat.

[83] Vinik AI, Ziegler D (2007). Diabetic cardiovascular autonomic neuropathy. Circulation.

[84] Du Maddaloni E, Moretti C, Del Toro R, Sterpetti S, Ievolella MV, Arnesano G (2021). Risk of cardiac autonomic neuropathy in latent autoimmune diabetes in adults is similar to type 1 diabetes and lower compared to type 2 diabetes: a cross-sectional study. Diabet Med.

[85] Debono M, Cachia E (2007). The impact of cardiovascular autonomic neuropathy in diabetes: is it associated with left ventricular dysfunction?. Auton Neurosci.

[86] Nathan DM, Genuth S, Lachin J, Cleary P, Crofford O, Davis M (1993). The effect of intensive treatment of diabetes on the development and progression of long-term complications in insulin-dependent diabetes mellitus. N Engl J Med.

[87] Pop-Busui R, Kirkwood I, Schmid H, Marinescu V, Schroeder J, Larkin D (2004). Sympathetic dysfunction in type 1 diabetes: association with impaired myocardial blood flow reserve and diastolic dysfunction. J Am Coll Cardiol.

[88] Sousa GR, Pober D, Galderisi A, Lv HJ, Yu L, Pereira AC (2019). Glycemic control, cardiac autoimmunity, and long-term risk of cardiovascular disease in type 1 diabetes mellitus. Circulation.

[89] Lv HJ, Havari E, Pinto S, Gottumukkala RVSRK, Cornivelli L, Raddassi K (2011). Impaired thymic tolerance to α-myosin directs autoimmunity to the heart in mice and humans. J Clin Investig.

[90] Xu X, Kobayashi S, Chen K, Timm D, Volden P, Huang Y (2013). Diminished autophagy limits cardiac injury in mouse models of type 1 diabetes. J Biol Chem.

[91] Kubli DA, Gustafsson ÅB (2015). Unbreak my heart: targeting mitochondrial autophagy in diabetic cardiomyopathy. Antioxid Redox Signal.

[92] Riehle C, Abel ED (2014). Insulin regulation of myocardial autophagy. Circ J.

[93] Xie Z, Lau K, Eby B, Lozano P, He C, Pennington B (2011). Improvement of cardiac functions by chronic metformin treatment is associated with enhanced cardiac autophagy in diabetic OVE26 mice. Diabetes.

[94] Ikeda Y, Shirakabe A, Maejima Y, Zhai P, Sciarretta S, Toli J (2015). Endogenous Drp1 mediates mitochondrial autophagy and protects the heart against energy stress. Circ Res.

[95] Zhao Y, Zhang L, Qiao Y, Zhou X, Wu G, Wang L (2013). Heme oxygenase-1 prevents cardiac dysfunction in streptozotocin-diabetic mice by reducing inflammation, oxidative stress. Apoptosis Enhanc Autophagy PLoS ONE.

[96] Lancel S, Montaigne D, Marechal X, Marciniak C, Hassoun SM, Decoster B (2012). Carbon monoxide improves cardiac function and mitochondrial population quality in a mouse model of metabolic syndrome. PLoS ONE.

[97] Guo R, Zhang Y, Turdi S, Ren J (2013). Adiponectin knockout accentuates high fat diet-induced obesity and cardiac dysfunction: role of autophagy. Biochem Biophys Acta.

[98] Cui M, Yu H, Wang J, Gao J, Li J (2013). Chronic caloric restriction and exercise improve metabolic conditions of dietary-induced obese mice in autophagy correlated manner without involving AMPK. J Diabet Res.

[99] Mellor KM, Bell JR, Young MJ, Ritchie RH, Delbridge LMD (2011). Myocardial autophagy activation and suppressed survival signaling is associated with insulin resistance in fructose-fed mice. J Mol Cell Cardiol.

[100] Russo SB, Baicu CF, Van Laer A, Geng T, Kasiganesan H, Zile MR (2012). Ceramide synthase 5 mediates lipid-induced autophagy and hypertrophy in cardiomyocytes. J Clin Investig.

[101] Pérez A, Wägner AM, Carreras G, Giménez G, Sánchez-Quesada JL, Rigla M (2000). Prevalence and phenotypic distribution of dyslipidemia in type 1 diabetes mellitus: effect of glycemic control. Arch Intern Med.

[102] Vinagre I, Sánchez-Quesada JL, Sánchez-Hernández J, Santos D, Ordoñez-Llanos J, De Leiva A (2014). Inflammatory biomarkers in type 2 diabetic patients: effect of glycemic control and impact of LDL subfraction phenotype. Cardiovasc Diabetol.

[103] Varbo A, Benn M, Nordestgaard BG (2014). Remnant cholesterol as a cause of ischemic heart disease: evidence, definition, measurement, atherogenicity, high risk patients, and present and future treatment. Pharmacol Ther.

[104] Velez M, Kohli S, Sabbah HN (2014). Animal models of insulin resistance and heart failure. Heart Fail Rev.

[105] Dunlay SM, Roger VL, Weston SA, Jiang R, Redfield MM (2012). Longitudinal changes in ejection fraction in heart failure patients with preserved and reduced ejection fraction. Circ Heart Fail.

[106] Seferović PM, Paulus WJ (2015). Clinical diabetic cardiomyopathy: a two-faced disease with restrictive and dilated phenotypes. Eur Heart J.

[107] McDonagh TA, Metra M, Adamo M, Baumbach A, Böhm M, Burri H (2021). 2021 ESC guidelines for the diagnosis and treatment of acute and chronic heart failure. Eur Heart J.

[108] Marx N, Federici M, Schütt K, Müller-Wieland D, Ajjan RA, Antunes MJ (2023). ESC guidelines for the management of cardiovascular disease in patients with diabetes. Eur Heart J.

[109] Alonso N, Lupón J, Barallat J, de Antonio M, Domingo M, Zamora E (2016). Impact of diabetes on the predictive value of heart failure biomarkers. Cardiovasc Diabetol.

[110] Tofte N, Theilade S, Winther SA, Birkelund S, Goetze JP, Hansen TW (2021). Comparison of natriuretic peptides as risk markers for all-cause mortality and cardiovascular and renal complications in individuals with type 1 diabetes. Diabet Care.

[111] Nagueh SF, Smiseth OA, Appleton CP, Byrd BF, Dokainish H, Edvardsen T (2016). Recommendations for the evaluation of left ventricular diastolic function by echocardiography: an update from the american society of echocardiography and the european association of cardiovascular imaging. J Am Soc Echocardiogr.

[112] Murtaza G, Virk HUH, Khalid M, Lavie CJ, Ventura H, Mukherjee D (2019). Diabetic cardiomyopathy—a comprehensive updated review. Prog Cardiovasc Dis.

[113] Kaushik A, Kapoor A, Dabadghao P, Khanna R, Kumar S, Garg N (2021). Use of strain, strain rate, tissue velocity imaging, and endothelial function for early detection of cardiovascular involvement in young diabetics. Ann Pediatr Cardiol.

[114] Seferović PM, Petrie MC, Filippatos GS, Anker SD, Rosano G, Bauersachs J (2018). Type 2 diabetes mellitus and heart failure: a position statement from the heart failure association of the european society of cardiology. Eur J Heart Fail.

[115] Heidenreich PA, Bozkurt B, Aguilar D, Allen LA, Byun JJ, Colvin MM (2022). 2022 AHA/ACC/HFSA guideline for the management of heart failure: a report of the American College of Cardiology/American Heart Association Joint Committee on clinical practice guidelines. Circulation.

[116] Elsayed NA, Aleppo G, Aroda VR, Bannuru RR, Brown FM, Bruemmer D (2023). Introduction and methodology: standards of care in diabetes—2023. Diabet Care.

[117] Yusuf S, Pitt B, Davis CE, Hood WB, Cohn JN (1991). Effect of enalapril on survival in patients with reduced left ventricular ejection fractions and congestive heart failure. N Engl J med.

[118] Young JB, Dunlap ME, Pfeffer MA, Probstfield JL, Cohen-Solal A, Dietz R (2004). Mortality and morbidity reduction with Candesartan in patients with chronic heart failure and left ventricular systolic dysfunction: results of the CHARM low-left ventricular ejection fraction trials. Circulation.

[119] MacMahon S, Sharpe N (1997). Randomised, placebo-controlled trial of carvedilol in patients with congestive heart failure due to ischaemic heart disease. Lancet.

[120] Hjalmarson Å, Goldstein S, Fagerberg B, Wedel H, Waagstein F, Kjekshus J (2000). Effects of controlled-release metoprolol on total mortality, hospitalizations, and well-being in patients with heart failure: the metoprolol CR/XL randomized Intervention trial in congestive heart failure (MERIT-HF). MERIT-HF Study Group JAMA.

[121] Pitt B, Zannad F, Remme WJ, Cody R, Castaigne A, Perez A (1999). The effect of spironolactone on morbidity and mortality in patients with severe heart failure. randomized aldactone evaluation study investigators. N Engl J med.

[122] Zannad F, McMurray JJV, Krum H, van Veldhuisen DJ, Swedberg K, Shi H (2011). Eplerenone in patients with systolic heart failure and mild symptoms. N Engl J Med.

[123] McMurray JJ, Packer M, Desai AS, Gong J, Lefkowitz MP, Rizkala AR (2014). Angiotensin–neprilysin inhibition versus enalapril in heart failure. N Eng J med.

[124] Swedberg K, Komajda M, Böhm M, Borer JS, Ford I, Dubost-Brama A (2010). Ivabradine and outcomes in chronic heart failure (SHIFT): a randomised placebo-controlled study. Lancet (London, Eng).

[125] Zinman B, Lachin JM, Inzucchi SE. Empagliflozin, Cardiovascular Outcomes, and Mortality in Type 2 Diabetes. N Engl J Med. 2016; 374:1094. 10.1056/NEJMc1600827. 10.1056/NEJMc1600827 26981940

[126] Rajagopalan S, Brook R (2017). Canagliflozin and cardiovascular and renal events in type 2 diabetes. N Engl J Med.

[127] Wiviott SD, Raz I, Bonaca MP, Mosenzon O, Kato ET, Cahn A (2019). Dapagliflozin and cardiovascular outcomes in type 2 diabetes. N Engl J Med.

[128] Cannon CP, Pratley R, Dagogo-Jack S, Mancuso J, Huyck S, Masiukiewicz U (2020). Cardiovascular outcomes with ertugliflozin in type 2 diabetes. N Engl J Med.

[129] McGuire DK, Shih WJ, Cosentino F, Charbonnel B, Cherney DZI, Dagogo-Jack S (2021). Association of sglt2 inhibitors with cardiovascular and kidney outcomes in patients with type 2 diabetes: a meta-analysis. JAMA Cardiol.

[130] Solomon SD, McMurray JJV, Claggett B, de Boer RA, DeMets D, Hernandez AF (2022). Dapagliflozin in heart failure with mildly reduced or preserved ejection fraction. N Engl J Med.

[131] Packer M, Anker SD, Butler J, Filippatos G, Pocock SJ, Carson P (2020). Cardiovascular and renal outcomes with empagliflozin in heart failure. N Engl J Med.

[132] Anker SD, Butler J, Filippatos G, Ferreira JP, Bocchi E, Böhm M (2021). Empagliflozin in heart failure with a preserved ejection fraction. N Engl J Med.

[133] Bhatt DL, Szarek M, Steg PG, Cannon CP, Leiter LA, McGuire DK (2021). Sotagliflozin in patients with diabetes and recent worsening heart failure. N Engl J Med.

[134] Voors AA, Angermann CE, Teerlink JR, Collins SP, Kosiborod M, Biegus J (2022). The SGLT2 inhibitor empagliflozin in patients hospitalized for acute heart failure: a multinational randomized trial. Nat Med.

[135] Bethel MA, Patel RA, Merrill P, Lokhnygina Y, Buse JB, Mentz RJ (2018). Cardiovascular outcomes with glucagon-like peptide-1 receptor agonists in patients with type 2 diabetes: a meta-analysis. Lancet Diabet Endocrinol.

[136] Kristensen SL, Rørth R, Jhund PS, Docherty KF, Sattar N, Preiss D (2019). Cardiovascular, mortality, and kidney outcomes with GLP-1 receptor agonists in patients with type 2 diabetes: a systematic review and meta-analysis of cardiovascular outcome trials. Lancet Diabet Endocrinol.

[137] Kosiborod MN, Abildstrøm SZ, Borlaug BA, Butler J, Rasmussen S, Davies M (2023). Semaglutide in patients with heart failure with preserved ejection fraction and obesity. N Engl J med.

[138] Lincoff AM, Brown-Frandsen K, Colhoun HM, Deanfield J, Emerson SS, Esbjerg S (2023). Semaglutide and cardiovascular outcomes in obesity without diabetes. N Engl J Med.

[139] Hodrea J, Saeed A, Molnar A, Fintha A, Barczi A, Wagner LJ (2022). SGLT2 inhibitor dapagliflozin prevents atherosclerotic and cardiac complications in experimental type 1 diabetes. PLoS ONE.

[140] Evans M, Hicks D, Patel D, Patel V, McEwan P, Dashora U (2020). Optimising the benefits of sglt2 inhibitors for type 1 diabetes. Diabet Therapy.

[141] Dandona P, Mathieu C, Phillip M, Hansen L, Tschöpe D, Thorén F (2018). Efficacy and safety of dapagliflozin in patients with inadequately controlled type 1 diabetes: the DEPICT-1 52-week study. Diabet Care.

[142] Buse JB, Garg SK, Rosenstock J, Bailey TS, Banks P, Bode BW (2018). Sotagliflozin in combination with optimized insulin therapy in adults with type 1 diabetes: the North American Intandem1 study. Diabet Care.

[143] Danne T, Cariou B, Banks P, Brandle M, Brath H, Franek E (2018). HbA1c and hypoglycemia reductions at 24 and 52 weeks with sotagliflozin in combination with insulin in adults with type 1 diabetes: the European Intandem2 study. Diabet Care.

[144] Cavallari I, Maddaloni E, Pieralice S, Mulè MT, Buzzetti R, Ussia GP (2020). The vicious circle of left ventricular dysfunction and diabetes: from pathophysiology to emerging treatments. J Clin Endocrinol Metab.

[145] Holman RR, Paul SK, Bethel MA, Matthews DR, Neil HAW (2008). 10-year follow-up of intensive glucose control in type 2 diabetes. N Engl J Med.

[146] The Diabetes Control and Complications Trial (DCCT) Research Group (1995). Effect of intensive diabetes management on macrovascular events and risk factors in the diabetes control and complications trial. Am J cardiol.

[147] Nathan DM, Bayless M, Cleary P, Genuth S, Gubitosi-Klug R, Lachin JM (2013). Diabetes control and complications trial/epidemiology of diabetes interventions and complications study at 30 years: advances and contributions. Diabetes.

[148] Turner R (1998). Effect of intensive blood-glucose control with metformin on complications in overweight patients with type 2 diabetes (UKPDS 34). Lancet.

[149] Nathan DM, Genuth S, Lachin J, Cleary P, Crofford O, Davis M (1993). The effect of intensive treatment of diabetes on the development and progression of long-term complications in insulin-dependent diabetes mellitus. Engl J Med.

[150] Turner R (1998). Intensive blood-glucose control with sulphonylureas or insulin compared with conventional treatment and risk of complications in patients with type 2 diabetes (UKPDS 33). Lancet.

[151] Patel A, MacMahon S, Chalmers J, Neal B, Billot L, Woodward M (2008). Intensive blood glucose control and vascular outcomes in patients with type 2 diabetes. N Engl J Med.

[152] Ismail-Beigi F, Craven T, Banerji MA, Basile J, Calles J, Cohen RM (2010). Effect of intensive treatment of hyperglycaemia on microvascular outcomes in type 2 diabetes: an analysis of the ACCORD randomised trial. Lancet (London, England).

[153] Castagno D, Baird-Gunning J, Jhund PS, Biondi-Zoccai G, MacDonald MR, Petrie MC (2011). Intensive glycemic control has no impact on the risk of heart failure in type 2 diabetic patients: evidence from a 37,229 patient meta-analysis. Am Heart J.

[154] Jarnert C, Landstedt-Hallin L, Malmberg K, Melcher A, Ohrvik J, Persson H (2009). A randomized trial of the impact of strict glycaemic control on myocardial diastolic function and perfusion reserve: a report from the DADD (diabetes mellitus and diastolic dysfunction) study. Eur J Heart Fail.

[155] Von Bibra H, Hansen A, Dounis V, Bystedt T, Malmberg K, Rydén L (2004). Augmented metabolic control improves myocardial diastolic function and perfusion in patients with non-insulin dependent diabetes. Heart (British Cardiac Soc).

[156] Julián MT, Alonso N, Lupón J, Gavidia-Bovadilla G, Ferrer E, De Antonio M (2020). Long-term LVEF trajectories in patients with type 2 diabetes and heart failure: diabetic cardiomyopathy may underlie functional decline. Cardiovasc Diabetol.

[157] Standl E, Stevens SR, Lokhnygina Y, Angelyn Bethel M, Buse JB, Gustavson SM (2020). Confirming the bidirectional nature of the association between severe hypoglycemic and cardiovascular events in type 2 diabetes: insights from EXSCEL. Diabet Care.

[158] Standl E, Stevens SR, Armstrong PW, Buse JB, Chan JCN, Green JB (2018). Increased risk of severe hypoglycemic events before and after cardiovascular outcomes in TECOS suggests an at-risk type 2 diabetes frail patient phenotype. Diabet Care.

[159] Pratley RE, Husain M, Lingvay I, Pieber TR, Mark T, Saevereid HA (2019). Heart failure with insulin degludec versus glargine U100 in patients with type 2 diabetes at high risk of cardiovascular disease: DEVOTE 14. Cardiovasc Diabetol.

[160] Fitchett D, Inzucchi SE, Wanner C, Mattheus M, George JT, Vedin O (2020). Relationship between hypoglycaemia, cardiovascular outcomes, and empagliflozin treatment in the EMPA-REG OUTCOME^®^ trial. Eur Heart J.

[161] Das SR, Drazner MH, Yancy CW, Stevenson LW, Gersh BJ, Dries DL (2004). Effects of diabetes mellitus and ischemic heart disease on the progression from asymptomatic left ventricular dysfunction to symptomatic heart failure: a retrospective analysis from the studies of left ventricular dysfunction (SOLVD) prevention trial. Am Heart J.

[162] Elder DHJ, Singh JSS, Levin D, Donnelly LA, Choy AM, George J (2016). Mean HbA1c and mortality in diabetic individuals with heart failure: a population cohort study. Eur J Heart Fail.

[163] Gruden G, Barutta F, Chaturvedi N, Schalkwijk C, Stehouwer CD, Witte DR (2012). Severe hypoglycemia and cardiovascular disease incidence in type 1 diabetes: the EURODIAB prospective complications study. Diabet Care..

[164] Lu CL, Shen HN, Hu SC, Der WJ, Li CY (2016). A population-based study of all-cause mortality and cardiovascular disease in association with prior history of hypoglycemia among patients with type 1 diabetes. Diabet Care..

[165] Giménez M, López JJ, Castell C, Conget I (2012). Hypoglycaemia and cardiovascular disease in type 1 diabetes results from the catalan national public health registry on insulin pump therapy. Diabet Res Clin Pract.

[166] Khunti K, Davies M, Majeed A, Thorsted BL, Wolden ML, Paul SK (2015). Hypoglycemia and risk of cardiovascular disease and all-cause mortality in insulin-treated people with type 1 and type 2 diabetes: a cohort study. Diabet Care.

[167] McCoy RG, Shah ND, Van Houten HK, Wermers RA, Ziegenfuss JY, Smith SA (2012). Increased mortality of patients with diabetes reporting severe hypoglycemia. Diabet Care..

[168] Colette C, Monnier L (2007). Acute glucose fluctuations and chronic sustained hyperglycemia as risk factors for cardiovascular diseases in patients with type 2 diabetes. Horm Metab Res.

[169] Xia J, Hu S, Xu J, Hao H, Yin C, Xu D (2018). The correlation between glucose fluctuation from self-monitored blood glucose and the major adverse cardiac events in diabetic patients with acute coronary syndrome during a 6-month follow-up by wechat application. Clin Chem Lab Med.

[170] Saisho Y (2014). Glycemic variability and oxidative stress: a link between diabetes and cardiovascular disease?. Int J Mol Sci.

[171] Martinez M, Santamarina J, Pavesi A, Musso C, Umpierrez GE (2021). Glycemic variability and cardiovascular disease in patients with type 2 diabetes. BMJ Open Diabet Res Care.

[172] Helleputte S, De Backer T, Lapauw B, Shadid S, Celie B, Van Eetvelde B (2020). The relationship between glycaemic variability and cardiovascular autonomic dysfunction in patients with type 1 diabetes: a systematic review. Diabetes/metabolism Res Rev.

[173] Alfieri V, Myasoedova VA, Vinci MC, Rondinelli M, Songia P, Massaiu I (2021). The role of glycemic variability in cardiovascular disorders. Int J mol sci.

[174] Phillip M, Nimri R, Bergenstal RM, Barnard-Kelly K, Danne T, Hovorka R (2023). Consensus recommendations for the use of automated insulin delivery technologies in clinical practice. Endocr Rev.

[175] Pauley ME, Tommerdahl KL, Snell-Bergeon JK, Forlenza GP (2022). Continuous glucose monitor, insulin pump, and automated insulin delivery therapies for type 1 diabetes: an update on potential for cardiovascular benefits. Curr Cardiol Rep.

[176] Kamrath C, Tittel SR, Kapellen TM, von dem Berge T, Heidtmann B, Nagl K (2021). Early versus delayed insulin pump therapy in children with newly diagnosed type 1 diabetes: results from the multicentre, prospective diabetes follow-up DPV registry. Lancet Child Adolesc Health.

[177] Derosa G, Catena G, Scelsi L, D’Angelo A, Raddino R, Cosentino E (2020). Glyco-metabolic control, inflammation markers, and cardiovascular outcomes in type 1 and type 2 diabetic patients on insulin pump or multiple daily injection (italico study). Diabetes/metabolism Res Rev.

[178] Steineck I, Cederholm J, Eliasson B, Rawshani A, Eeg-Olofsson K, Svensson AM (2015). Insulin pump therapy, multiple daily injections, and cardiovascular mortality in 18,168 people with type 1 diabetes: observational study. BMJ (Clin Research Ed)..

[179] Giménez-Pérez G, Viñals C, Mata-Cases M, Vlacho B, Real J, Franch-Nadal J (2023). Epidemiology of the first-ever cardiovascular event in people with type 1 diabetes: a retrospective cohort population-based study in catalonia. Cardiovasc Diabetol.

[180] Larsson SC, Wallin A, Håkansson N, Stackelberg O, Bäck M, Wolk A (2018). Type 1 and type 2 diabetes mellitus and incidence of seven cardiovascular diseases. Int J Cardiol.

